# Risk Factors, Clinical and Endoscopic Features, and Clinical Outcomes in Patients with Cytomegalovirus Esophagitis

**DOI:** 10.3390/jcm11061583

**Published:** 2022-03-13

**Authors:** Pai-Jui Yeh, Ren-Chin Wu, Chien-Ming Chen, Cheng-Tang Chiu, Ming-Wei Lai, Chien-Chang Chen, Chia-Jung Kuo, Jun-Te Hsu, Ming-Yao Su, Puo-Hsien Le

**Affiliations:** 1 Department of Pediatric Gastroenterology, Chang Gung Memorial Hospital, Linkou Branch, Taoyuan 333, Taiwan; charlie01539@hotmail.com (P.-J.Y.); a22141@cgmh.org.tw (M.-W.L.); cgj2841@cgmh.org.tw (C.-C.C.); 2 Department of Pathology, Chang Gung Memorial Hospital, Linkou Branch, Taoyuan 333, Taiwan; renchin.wu@gmail.com; 3 Department of Medical Imaging and Interventions, Chang Gung Memorial Hospital, Linkou Branch, Taoyuan 333, Taiwan; dr.cmchen@gmail.com; 4 Department of Gastroenterology and Hepatology, Chang Gung Memorial Hospital, Linkou Branch, Taoyuan 333, Taiwan; ctchiu@cgmh.org.tw (C.-T.C.); m7011@cgmh.org.tw (C.-J.K.); 5 Taiwan Association of the Study of Small Intestinal Disease, Taoyuan 333, Taiwan; doctorsu@cgmh.org.tw; 6 Liver Research Center, Chang Gung Memorial Hospital, Linkou Branch, Taoyuan 333, Taiwan; 7 Department of General Surgery, Chang Gung Memorial Hospital, Linkou Branch, Taoyuan 333, Taiwan; hsujt2813@cgmh.org.tw; 8 Department of Gastroenterology and Hepatology, New Taipei City Municipal Tucheng Hospital, New Taipei City 236, Taiwan

**Keywords:** acute kidney injury, cytomegalovirus, endoscopy, esophagitis, prognostic factor

## Abstract

Cytomegalovirus (CMV) esophagitis is the second most common CMV disease of the gastrointestinal tract. This study aims to comprehensively analyze risk factors, clinical characteristics, endoscopic features, outcomes, and prognostic factors of CMV esophagitis. We retrospectively collected data of patients who underwent esophageal CMV immunohistochemistry (IHC) staining between January 2003 and April 2021 from the pathology database at the Chang Gung Memorial Hospital. Patients were divided into the CMV and non-CMV groups according to the IHC staining results. We enrolled 148 patients (44 CMV and 104 non-CMV patients). The risk factors for CMV esophagitis were male sex, immunocompromised status, and critical illness. The major clinical presentations of CMV esophagitis included epigastric pain (40.9%), fever (36.4%), odynophagia (31.8%), dysphagia (29.5%), and gastrointestinal bleeding (29.5%). Multiple diffuse variable esophageal ulcers were the most common endoscopic feature. The CMV group had a significantly higher in-hospital mortality rate (18.2% vs. 0%; *p* < 0.001), higher overall mortality rate (52.3% vs. 14.4%; *p* < 0.001), and longer admission duration (median, 24 days (interquartile range (IQR), 11–47 days) vs. 14 days (IQR, 7–24 days); *p* = 0.015) than the non-CMV group. Acute kidney injury (odds ratio (OR), 174.15; 95% confidence interval (CI), 1.27–23,836.21; *p* = 0.040) and intensive care unit admission (OR, 26.53; 95% CI 1.06–665.08; *p* = 0.046) were predictors of in-hospital mortality. In conclusion, the mortality rate of patients with CMV esophagitis was high. Physicians should be aware of the clinical and endoscopic characteristics of CMV esophagitis in high-risk patients for early diagnosis and treatment.

## 1. Introduction

Cytomegalovirus (CMV) esophagitis is the second most common CMV disease of the gastrointestinal tract and the third leading cause of infectious esophagitis [1,2]. It is usually diagnosed in immunocompromised patients but is also seen in immunocompetent patients [3,4]. Because of the limited number of cases, most studies discussed CMV disease of the upper gastrointestinal tract or infectious esophagitis instead of focusing on CMV esophagitis [3,5,6]. The largest case series of CMV esophagitis enrolled 25 patients and narrated the clinical characteristics and endoscopic features of CMV esophagitis [3]. No study has investigated the risk factors and prognostic factors of CMV esophagitis. This is the first retrospective cohort study to provide comprehensive information on CMV esophagitis, including risk factors, clinical characteristics, endoscopic features, treatments, outcomes, and prognostic factors.

## 2. Materials and Methods

### 2.1. Compliance with Ethical Standards

The study protocol was approved by the Institutional Review Board (IRB) of the Chang Gung Medical Foundation (approval document No. 202101234B0. “Clinical presentations and outcome of cytomegalovirus, herpes simplex virus, Epstein-Barr virus, and clostridioides infection”) for the period from 28 July 2021 to 27 July 2022. Due to the retrospective nature of the study, the IRB waived the requirement of signed informed consent from individual patients to review medical records from the electronic medical record system. The study protocol conformed to the ethical guidelines of the 1975 Declaration of Helsinki, as reflected in the prior approval by the institution’s human research committee.

### 2.2. Patients

**Figure 1:**
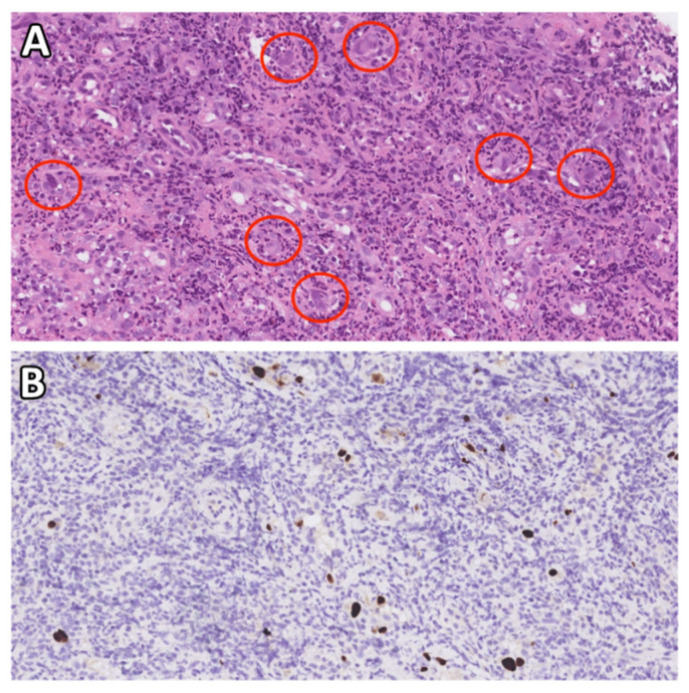
Diagnosis of CMV esophagitis using CMV IHC staining and/or CMV inclusion bodies in H&E staining. (**A**) H&E staining (40× objective) showing typical intranuclear (owl’s eye) and intracytoplasmic (eosinophilic punctiform) CMV inclusions within the circles. (**B**) IHC staining (40× objective) with 1:200 diluted Novocastra™ lyophilized mouse monoclonal antibody against CMV pp65 antigen shows strong focal CMV immunoreactivity with brownish areas. CMV—cytomegalovirus; H&E—hematoxylin and eosin; IHC—immunohistochemistry.

In this retrospective cohort study, we enrolled all patients with esophageal CMV immunohistochemistry (IHC) staining results from the pathology database at the Linkou Chang Gung Memorial Hospital who underwent esophageal CMV immunohistochemistry (IHC) staining between January 2003 and April 2021. Patients were divided into the CMV and non-CMV groups according to the IHC staining results. CMV esophagitis was diagnosed based on positive CMV IHC staining of the esophageal tissue, with or without viral inclusion bodies, using hematoxylin and eosin staining (Figure 1). CMV IHC was performed using monoclonal antibodies directed against the CMV pp65 antigen (Novocastra™ lyophilized mouse monoclonal antibody; Leica Microsystems, Wetzlar, Germany).

### 2.3. Data Collection

Medical records of eligible patients were reviewed for data on age; sex; patient source (outpatient or inpatient); admission duration; date of diagnosis (the date of final pathological confirmation); acquisition time (interval between the date of admission or outpatient clinic visit to the date of diagnosis); recurrence; death or last follow-up; presence of critical conditions, such as shock, pneumonia, and respiratory distress requiring intubation within 1 week before diagnosis; requirement of intensive care unit (ICU) admission; underlying disease; medication history; major clinical presentation; endoscopic findings including lesion characteristics, location, number, and concomitant mucosal findings; histopathological results including presence of malignancy or findings indicating other etiology of esophagitis; results of other imaging tests such as upper gastrointestinal series study and computed tomography; treatment and therapeutic duration; complications; outcomes including admission duration, in-hospital mortality rate, and overall mortality rate; hematological parameters including total white blood cell count, absolute neutrophil count, absolute lymphocyte count, platelet count, and hemoglobin level; biochemical parameters including creatinine, aspartate aminotransferase, alanine aminotransferase, bilirubin, albumin, and C-reactive protein (CRP) levels; CMV pp65 antigenemia; CMV DNA ( Light-Mix^®^ Kit human cytomegalovirus (TIB Molbiol, Berlin, Germany, cut-off: Cp 35, 226 bp segment on glycoprotein B gene), COBAS^®^ AmpliPrep/COBAS^®^ TaqMan^®^ CMV Test (Roche Diagnostics, Branchburg, NJ, USA, cut-off: 150 copies/mL)); and CMV serology.

### 2.4. Definitions

The locations of esophageal lesions (upper, middle, and lower third) were categorized according to the cancer staging manual of the American Joint Committee on Cancer [7]. Barrett’s esophagus was diagnosed according to the American College of Gastroenterology guideline [8]. Patients were defined as “immunocompromised” if they were documented to have primary immunodeficiency, human immunodeficiency virus (HIV) infection, underlying malignancy with exposure to radiotherapy or chemotherapeutic agents within 6 months, use of immunosuppressants including corticosteroids (oral or intravenous administration ≥20 mg/day of prednisolone or any equivalent for >2 weeks), or were recipients of solid organ or bone marrow transplantation [9,10].

### 2.5. Statistical Analyses

Numerical data are presented as mean ± standard deviation or median (interquartile range [IQR]), while categorical data are expressed as absolute numbers and percentages. An independent *t*-test or Mann–Whitney U test was used to compare continuous variables, while the χ^2^ and Fisher’s exact tests were used for categorical variables. Logistic regression models were used to identify independent risk factors for in-hospital mortality. Statistical significance was set at *p* < 0.05. The results are presented as odds ratios (ORs), 95% confidence intervals (CIs), and *p*-values. Survival outcomes were evaluated using the Kaplan–Meier survival curve analysis and log-rank test. All statistical calculations were performed using SPSS statistical software (version 21.0; IBM Corp., Armonk, NY, USA).

## 3. Results

### 3.1. Baseline Characteristics of Patients with CMV Esophagitis

A total of 148 patients were enrolled, including 44 and 104 patients in the CMV and non-CMV groups, respectively. The CMV group was predominated by men (77.3%), with a mean age of 59.5 ± 18.5 years. Three-quarters were hospitalized, and 15.9% required ICU care. Up to 77.3% of this group was considered immunocompromised, and the major underlying diseases were malignancy, gastroesophageal reflux disease (GERD), and hypertension. Most malignancies in the CMV group were located in the neck and chest areas, including seven esophageal cancers, six lung cancers, two breast cancers, and one orbital melanoma with thoracic spine metastasis. All 16 patients in this group underwent radiation therapy and had radiation exposure in the region of the esophagus. HIV infection, solid organ transplant (kidney), and autoimmune disease (systemic lupus erythematosus) were noted in eight, three, and two patients, respectively. No patient with Crohn’s disease or prior esophageal surgery was noted in this study. Seven patients had other CMV diseases, including CMV gastritis (three patients), extra-alimentary diseases (three patients; two patients with hepatitis and one patient with retinitis), and one patient with both retinitis and gastritis. The most common baseline medications were antibiotics (75%), proton pump inhibitors (PPIs) (63.6%), and steroids (52.3%). Other details are listed in Table 1.

### 3.2. Clinical Manifestations of CMV Esophagitis

The most common symptoms were epigastric pain (40.9%), fever (36.4%), odynophagia (31.8%), dysphagia (29.5%), and gastrointestinal bleeding (29.5%). No patient developed esophageal perforation, fistula, stricture, or mediastinitis.

### 3.3. Results of Laboratory Examinations in Patients with CMV Esophagitis

Anemia, hypoalbuminemia, and elevated CRP levels indicated a poor general condition with inflammation/infection in patients with CMV esophagitis. Although CMV serology tests were valuable, the data were incomplete for a real-world study. According to available data, the positivity rates of CMV IgM, IgG, antigenemia, and viremia were 21.4% (3/14), 92.9% (13/14), 66.7% (8/12), and 57.1% (4/7), respectively.

### 3.4. Endoscopic Findings in Patients with CMV Esophagitis

**Figure 2:**
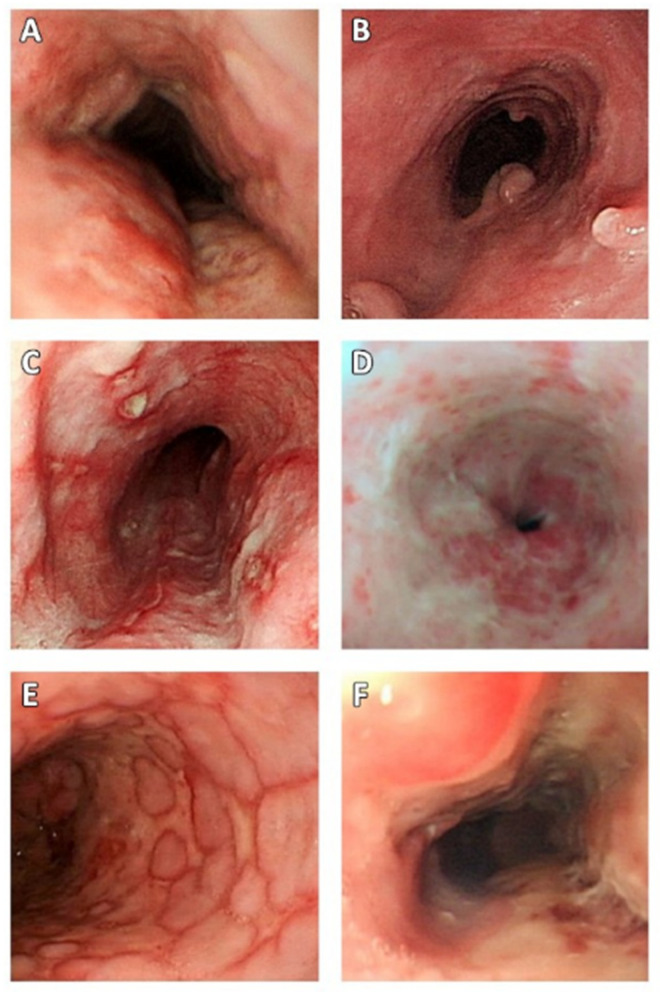
Endoscopic features of CMV esophagitis. (**A**) Inflammation; (**B**) polypoid lesion; (**C**–**F**) variable morphologies of esophageal ulcers. CMV—cytomegalovirus.

All specimens in the study were obtained from endoscopic biopsies without surgical resection. With regard to endoscopic features, diffuse and multiple (≥2) ulcers with variable morphologies and sizes were the most common finding (Figure 2). The lower (86.4%) and middle (61.4%) esophagus were commonly involved areas. Reflux esophagitis, Barrett’s esophagus, and esophageal candidiasis were noted in 38.6%, 9.1%, and 18.2% of patients, respectively. Moreover, 29.5% of the patients had concurrent gastric ulcers. However, no malignant cells were observed in any specimen.

### 3.5. Radiological Examination of Patients with CMV Esophagitis

In the CMV group, an upper gastrointestinal series was performed for two patients, and nine patients underwent computed tomography for underlying diseases but not for CMV esophagitis. These findings were not related to CMV esophagitis.

### 3.6. Treatments and Outcomes in Patients with CMV Esophagitis

The median acquisition time was 8 days (IQR, 5–14 days). After diagnosis, 26 (59.1%) patients were administered antiviral treatment (valganciclovir, oral form; ganciclovir, intravenous or oral form), and 31 (70.5%) patients were prescribed PPIs. None of the patients required surgical intervention for CMV-related complications. The median admission duration was 24 days (IQR, 11–47 days). The in-hospital and overall mortality rates were 18.2% and 52.3%, respectively. The median follow-up duration was 276 days (IQR, 64.8–738.8 days) days, and no disease recurrence was observed.

### 3.7. Differences between the CMV and Non-CMV Groups

With regard to risk factors, there was a significantly higher male to female ratio; more inpatients; more immunocompromised patients, including patients with HIV infection, malignancy, organ transplant, steroid usage, chemotherapy, and radiotherapy; and more critically ill patients, including patients with shock, pneumonia, respiratory failure, and ICU requirement in the CMV group. Antibiotics, PPIs, other antacids, and mucosal protectants were more commonly used in the CMV group before the diagnosis of CMV disease. Clinically, CMV esophagitis was more likely to present with fever, hematemesis, gastrointestinal bleeding, odynophagia, and abdominal fullness. Laboratory examination revealed that patients with CMV esophagitis had lower blood cellular counts, lower albumin levels, and higher ALT levels. These findings suggested poor nutritional status and more severe infection. Regarding endoscopic features, upper and lower esophageal involvement, diffuse/multiple esophageal ulcers, concurrent esophageal candidiasis, and gastric ulcers were more common in the CMV group. Regarding clinical outcomes, patients with CMV esophagitis had a longer hospital stay and higher in-hospital and overall mortality rates.

### 3.8. Prognostic Factors for In-Hospital Mortality in Patients with CMV Esophagitis

**Figure 3:**
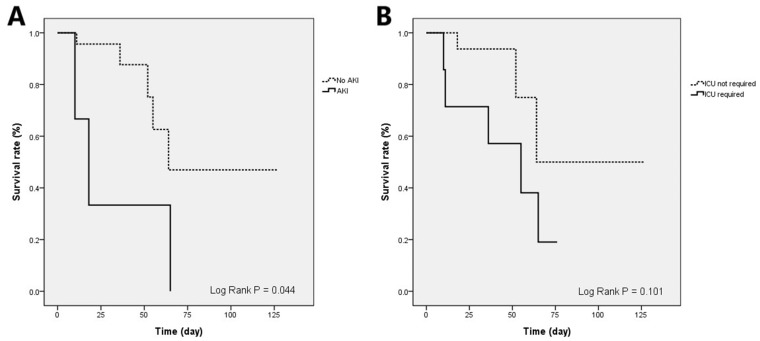
Kaplan–Meier survival curve analysis of patients with CMV esophagitis. (**A**) Patients with AKI (solid line) had a significantly worse survival rate than those without AKI (dash line) (log-rank *p* = 0.044). (**B**) Patients who required ICU care had a worse survival rate, although it was not statistically significant (log-rank *p* = 0.101). AKI—acute kidney injury; CMV—cytomegalovirus; ICU—intensive care unit.

Acute kidney injury (AKI) (OR, 174.148; 95% CI, 1.272–23,836.208; *p* = 0.04) and ICU requirement (OR, 26.526; 95% CI, 1.058–665.083; *p* = 0.046) were independent prognostic factors for in-hospital mortality (Table 2). In the Kaplan–Meier survival curve analysis, patients with AKI showed significantly worse survival rates than those without AKI (log-rank *p* = 0.044) (Figure 3).

## 4. Discussion

CMV esophagitis affects not only immunocompromised patients but also immunocompetent hosts, although it is predominant and leads to worse outcomes in the former. It is important to understand the risk factors and other information for early diagnosis and treatment, but related studies are limited. Most studies on CMV esophagitis have focused on patients with organ transplantation or HIV infection, [4,11,12,13] and provided clinical presentations and endoscopic findings in these groups of patients. Regarding the general population, the largest and latest case series enrolled 25 patients with CMV esophagitis, of which 4% were immunocompetent [3]. This report presented the clinical features, endoscopic findings, and clinical outcomes of patients with CMV esophagitis. However, it was difficult to identify specific risk factors, clinical characteristics, and endoscopic features without a control group. Therefore, we conducted the largest retrospective cohort study with 148 patients to provide comprehensive information on CMV esophagitis, from risk factors to clinical outcomes.

In this cohort study, we found that male sex, immunocompromised status, and critical illness were risk factors for CMV esophagitis. Many esophageal diseases, including GERD, Barrett’s esophagus, and esophageal adenocarcinoma, also exhibit male predominance [14,15]. This might be attributed to lifestyle, dietary habits, genes, or estrogen. Estrogen contributes to esophageal epithelial resistance to causative insults and modulates cytokine-induced barrier dysfunction, thereby decreasing the risk of esophageal epithelial infection [15,16,17]. As mentioned in previous reports, immunocompromised status, including concurrent chemoradiotherapy, increases the risk of CMV esophagitis because of leukopenia and mucosal defects [1,18]. Critical illness (shock and respiratory failure) leads to multiple organ dysfunction, hypoxia, and hypoperfusion; it is also regarded as an immunodeficient condition and is, therefore, a risk factor for CMV esophagitis. Regarding symptoms and endoscopic findings, when inpatients have fever, hematemesis, odynophagia, diffuse or multiple esophageal ulcers, and esophageal candidiasis, physicians should perform biopsy and CMV IHC staining to rule out CMV esophagitis.

There were 20 cases (45.5%) with malignant diseases in the CMV group, which was higher than in the non-CMV group. Among these patients, 80% of them had received radiation therapy, and all of them had radiation exposure in the area of the esophagus. Additionally, 75% of patients had received chemotherapy. Therefore, there were three possible explanations for the association between malignancies and CMV esophagitis: (1) chemotherapy suppresses immunity and substantially increases the risk of opportunistic infections; CMV reactivation in patients with esophageal cancer has been reported [18,19,20]; (2) local radiotherapy leads to mucosal injury and increases the risk of invasive CMV diseases [1,21]; (3) some studies noted the role of CMV in the pathogenesis of esophageal and other cancers, including immune escapes, tumor microenvironment, and oncomodulation [22,23].

ICU requirement and AKI were independent prognostic factors for in-hospital mortality. Patients with AKI also had poorer Kaplan–Meier survival curves. ICU admission suggests that the patients had severe disease activity and critical conditions, leading to a higher mortality rate. In our previous study of CMV gastritis, AKI was also a negative prognostic factor [24]. In critically ill patients, AKI is associated with higher ICU admission and mortality rates [25]. Furthermore, renal injury leads to T-cell exhaustion and dysfunction, resulting in imbalanced immune regulation and impaired clearance of pathogens [26,27]. Both perspectives were possible explanations for AKI being a prognostic factor for in-hospital mortality. Therefore, we should be alert to the risk factors, symptoms, and endoscopic features of CMV esophagitis and prevent AKI by avoiding agents causing renal toxicity and administering adequate hydration to improve the in-hospital mortality rate. Although some studies mentioned that antiviral therapies improved the in-hospital survival in cytomegalovirus diseases of the whole gastrointestinal tract, it showed no significant benefit for in-hospital mortality in this study [10,28]. This could be caused by a small case number, the heterogeneity of treatment course, immunity, variable disease severity, and the side effects of antiviral agents. A prospective study with a larger case number will help to clarify this issue.

In this largest cohort study of CMV esophagitis, we applied strict diagnostic criteria and comprehensively presented risk factors, clinical presentations, laboratory findings, endoscopic features, treatments, prognostic factors, and outcomes. Because we only enrolled the patients with IHC staining results and most biopsies were performed due to endoscopic findings, selection bias might have existed. Other limitations of this study included a single-center, retrospective study design and a relatively small number of cases.

## 5. Conclusions

Physicians should watch for the clinical and endoscopic characteristics of CMV esophagitis in high-risk patients for early diagnosis and treatment. In the therapeutic course, the prevention of AKI is important for improving the in-hospital mortality rate.

## Figures and Tables

**Table 1:** Baseline characteristics of patients with and without CMV esophagitis.

Characteristics	Overall (*n* = 148)	CMV Esophagitis (*n* = 44)	Non-CMV Esophagitis (*n* = 104)	*p*-Value
Age, years	56.7 ± 18.9	59.5 ± 18.5	56.9 ± 19.1	0.489
Gender (M/F)	96 (64.9%)/52 (35.1%)	34 (77.3%)/10 (22.7%)	62 (59.6%)/42 (40.4%)	0.040 *
OPD patients	71 (48%)	11 (25%)	60 (57.7%)	<0.001 *
Acquisition time (day)	8 (5–14)	11.5 (6.8–26)	7 (4–12)	0.005 *
General conditions				
Shock	10 (6.8%)	8 (18.2%)	2 (1.9%)	0.001 *
Pneumonia	25 (16.9%)	16 (36.4%)	9 (8.7%)	<0.001 *
Intubation	9 (6.1%)	6 (13.6%)	3 (2.9%)	0.02 *
ICU required	10 (6.8%)	7 (15.9%)	3 (3%)	0.008 *
Immunocompromised	68 (45.9%)	34 (77.3%)	34 (32.7%)	<0.001 *
Underlying diseases				
Diabetes mellitus	29 (19.6%)	8 (18.2%)	21 (20.2%)	0.78
Hypertension	54 (36.5%)	19 (43.2%)	35 (33.7%)	0.27
Autoimmune disease	7 (4.7%)	2 (4.5%)	5 (4.8%)	1
Crohn’s disease	1 (0.6%)	0 (0%)	1 (1%)	1
Ulcerative colitis	2 (1.4%)	1 (2.3%)	1 (1%)	0.51
Coronary artery disease	8 (5.4%)	5 (11.4%)	3 (2.9%)	0.05
COPD	8 (5.4%)	4 (9.1%)	4 (3.8%)	0.24
Renal disease				
AKI	13 (8.8%)	5 (11.4%)	8 (7.7%)	0.47
CKD	24 (16.2%)	7 (15.9%)	17 (16.3%)	0.95
ESRD	11 (7.4%)	4 (9.1%)	7 (6.7%)	0.73
HIV infection	11 (7.4%)	8 (18.2%)	3 (2.9%)	0.003 *
Malignancies	44 (29.7%)	20 (45.5%)	24 (23.1%)	0.01 *
Transplantation	3 (2%)	3 (6.8%)	0 (0%)	0.03 *
GERD	91 (61.5%)	20 (45.5%)	71 (68.3%)	0.01 *
Immunosuppressive therapies				
Steroid	35 (23.6%)	23 (52.3%)	12 (11.5%)	<0.001 *
Immunosuppressant	11 (7.4%)	6 (13.6%)	5 (4.8%)	0.06
Chemotherapy	27 (18.2%)	15 (34.1%)	12 (11.5%)	0.001 *
Radiotherapy	29 (19.6%)	16 (36.4%)	13 (12.5%)	0.001 *
Other medications				
Antibiotics	67 (45.3%)	33 (75%)	34 (32.7%)	<0.001 *
Proton pump inhibitor	75 (50.7%)	28 (63.6%)	47 (45.2%)	0.04 *
Other antacids	24 (16.2%)	14 (31.8%)	10 (9.6%)	0.001 *
Mucosal protectant	31 (20.9%)	14 (31.8%)	17 (16.3%)	0.03 *
Clinical presentation				
Fever	23 (15.5%)	16 (36.4%)	7 (6.7%)	<0.001*
Epigastric pain	48 (32.4%)	18 (40.9%)	30 (28.8%)	0.15
Vomiting	34 (23%)	11 (25%)	23 (22.1%)	0.70
Hematemesis	13 (8.8%)	9 (20.5%)	4 (3.8%)	0.002 *
GI bleeding †	29 (19.6%)	13 (29.5%)	16 (15.4%)	0.05 *
Dysphagia	37 (25%)	13 (29.5%)	24 (23.1%)	0.41
Odynophagia	27 (18.2%)	14 (31.8%)	13 (12.5%)	0.005 *
Abdominal fullness	13 (8.8%)	9 (20.5%)	4 (3.8%)	0.002 *
Laboratory data				
WBC count (/μL)	6900 (4250–9750)	4900 (2700–8575)	7600 (5200–10,300)	0.004 *
Hemoglobin (g/dL)	10.5 (9.2–12.1)	10.4 (9–11.6)	10.7 (9.4–13.2)	0.026 *
Platelets (×1000/mm^3^)	228 (136–284.5)	173.5 (116.5–250)	250 (183–295)	0.005 *
Creatinine (mg/dL)	0.9 (0.7–1.3)	0.73 (0.6–1.11)	0.9 (0.7–1.3)	0.071
ALT (IU/L)	20 (14–33)	24 (17.3–33)	17 (12–29)	0.022 *
Albumin (g/dL)	2.9 (2.5–3.7)	2.8 (2.3–2.9)	3.3 (2.8–4)	0.004 *
C-reactive protein (mg/dL)	32.4 (6.9–63.9)	43 (8.1–73.2)	29.2 (3.4–58.5)	0.415
Endoscopic features				
Main findings				
Ulcer	131 (88.5%)	39 (88.6%)	92 (88.5%)	0.98
Diffuse/multiple ulcers	90 (60.8%)	33 (75%)	57 (54.8%)	0.02 *
Inflammation	5 (3.4%)	1 (2.3%)	4 (3.8%)	1
Polypoid lesion	10 (6.8%)	6 (13.6%)	4 (3.8%)	0.07
Location of lesion				
Upper 3rd	31 (20.9%)	14 (31.8%)	17 (16.3%)	0.03 *
Middle 3rd	85 (57.4%)	27 (61.4%)	58 (55.8%)	0.53
Lower 3rd	110 (74.3%)	38 (86.4%)	72 (69.2%)	0.03 *
Concurrent findings				
Reflux esophagitis	75 (50.7%)	17 (38.6%)	58 (55.8%)	0.06
Esophageal candidiasis	12 (8.1%)	8 (18.2%)	4 (3.8%)	0.01 *
Barrett esophagus	9 (6.1%)	4 (9.1%)	5 (4.8%)	0.45
Gastric ulcer	25 (16.9%)	13 (29.5%)	12 (11.5%)	0.01 *
CMV gastritis	4 (2.7%)	4 (9.1%)	0 (0%)	0.01 *
CMV, others ‡	4 (2.7%)	4 (9.1%)	0 (0%)	0.01 *
Outcomes				
Follow-up duration (days)	351 (112.5–1187.3)	276 (64.8–738.8)	451.1 (141.3–1345.5)	0.198
Hospital stay (days)	17.5 (9–35.3)	24 (11–47)	14 (7–24)	0.02 *
In-hospital mortality	8 (5.4%)	8 (18.2%)	0 (0%)	<0.001 *
Overall mortality	38 (25.7%)	23 (52.3%)	15 (14.4%)	<0.001 *

* *p* < 0.05. Age is presented as mean ± standard deviation. Laboratory data, acquisition time, admission duration, and follow-up duration are presented as median (IQR). The remaining data are presented as numbers (percentages). † defined as hematemesis, tarry stool, or bloody stool. ‡ included two patients with CMV retinitis and two patients with CMV hepatitis. AKI—acute kidney injury; ALT—alanine aminotransferase; CKD—chronic kidney disease; CMV—cytomegalovirus; COPD—chronic obstructive pulmonary disease; ESRD—end-stage renal disease; F—female; GERD—gastroesophageal reflux disease; GI—gastrointestinal; HIV—human immunodeficiency virus; ICU—intensive care unit; IQR—interquartile range; M—male; OPD—outpatient department; SD—standard deviation; WBC—white blood cell.

**Table 2:** Analysis of clinical factors associated with in-hospital mortality in patients with CMV esophagitis.

Characteristics	Univariable Analysis			Multivariable Analysis		
	OR	95% CI	*p*-Value	OR	95% CI	*p*-Value
Age	1.04	0.99–1.09	0.11			
Gender (Male)	0.86	0.14–5.10	0.87			
Acquisition time	1.06	1.00–1.13	0.04 *			
General conditions						
Shock	18.33	2.87–117.33	0.002 *			
Intubation	17	2.33–124.19	0.01 *			
ICU required	28.33	3.76–213.70	0.001 *	26.53	1.06–665.08	0.05 *
Immunocompromised	0.86	0.14–5.10	0.87			
Underlying diseases						
Diabetes mellitus	1.67	0.27–10.33	0.58			
Hypertension	2.62	0.54–12.72	0.23			
Acute kidney injury	10.2	1.35–76.93	0.02 *	174.15	1.27–23,836.21	0.04 *
Chronic kidney disease	2.07	0.32–13.25	0.44			
End-stage renal disease	0	0–0	0.1			
Malignancy	1.25	0.27–5.80	0.78			
Chemotherapy	0.22	0.03–2.03	0.18			
Radiotherapy	1.06	0.22–5.18	0.94			
Steroid usage	1.67	0.35–8.04	0.53			
GERD	0.13	0.01–1.15	0.07			
CMV gastritis	5.67	0.66–48.33	0.11			
Clinical symptoms						
Fever	0.52	0.09–2.97	0.47			
Epigastric pain	2.95	0.61–14.38	0.18			
Vomiting	0.37	0.04–3.42	0.38			
Bloody vomiting	6.2	1.16–33.17	0.03 *			
GI bleeding	12.43	2.05–75.24	0.01 *			
Laboratory data						
WBC count	1	1–1	0.13			
Hemoglobin	0.77	0.48–1.21	0.25			
Platelet	0.10	0.99–1.01	0.76			
Creatinine	0.85	0.42–1.73	0.66			
ALT	1.04	0.99–1.09	0.09			
Albumin	0.21	0.04–1.05	0.06			
C-reactive protein	0.99	0.97–1.01	0.37			
Endoscopic features						
Diffuse/multiple ulcers	2.69	0.29–24.75	0.38			
Esophageal candidiasis	0.59	0.06–5.63	0.65			
Barrett’s esophagus	1.57	0.14–17.42	0.71			
Gastric ulcer	12.43	2.05–75.24	0.01 *			
Antiviral therapy	1.19	0.246–5.764	0.828			

* p < 0.05, calculated using logistic regression analysis. ALT—alanine aminotransferase; CI—confidence interval; CMV—cytomegalovirus; F—female; GERD—gastroesophageal reflux disease; GI—gastrointestinal; ICU—intensive care unit; M—male; OR—odds ratio; WBC—white blood cell.

## Data Availability

The data presented in this study are available on request from the corresponding author.
